# Biochars from Post-Production Biomass and Waste from Wood Management: Analysis of Carbonization Products

**DOI:** 10.3390/ma13214971

**Published:** 2020-11-04

**Authors:** Wojciech Kosakowski, Malgorzata Anita Bryszewska, Piotr Dziugan

**Affiliations:** 1 Polmos Żyrardów Sp. z o.o., ul. Mickiewicza 1-3, 96-300 Żyrardów, Poland; wkosakowski@belvederevodka.pl; 2 Institute of Natural Products and Cosmetics, Faculty of Biotechnology and Food Sciences, Lodz University of Technology, ul. Stefanowskiego 4/10, 90-924 Lodz, Poland; 3 Department of Environmental Biotechnology, Faculty of Biotechnology and Food Sciences, Lodz University of Technology, Wolczanska 171/173, 90-924 Lodz, Poland; piotr.dziugan@p.lodz.pl

**Keywords:** pyrolysis, biochars, agricultural waste, biomass

## Abstract

Waste biomass can be used as an alternative source of energy. However, such use requires prior treatment of the material. This paper describes the physicochemical characteristics of biochar obtained by the thermochemical decomposition of six types of agricultural waste biomass: residues from the production of flavored spirits (a pulp of lime, grapefruit and lemon), beetroot pulp, apple pomace, brewer’s spent grain, bark and municipal solid waste (bark, sawdust, off-cuts and wood chips). The biomass conversion process was studied under conditions of limited oxygen access in a reactor. The temperature was raised from 450 to 850 °C over 30 min, followed by a residence time of 60 min. The solid products were characterized in terms of their elemental compositions, mass, energy yield and ash content. The gaseous products from pyrolysis of the biomass were also analyzed and their compositions were characterized by GCMS (Gas Chromatography–Mass Spectrometry). The carbonization process increased the carbon content by, on average, 1.7 times, from an average percentage of 46.09% ± 3.65% for biomass to an average percentage of 74.72% ± 5.36% for biochars. After carbonization, the biochars were found to have a net calorific value of between 27 and 32 MJ/kg, which is comparable or even higher than good-quality coal (eco pea coal 24–26 MJ/kg). The net calorific values show that the volatile products can also be considered as a valuable source of energy.

## 1. Introduction

Recycling biomass and various types of organic waste is a way of increasing the share of renewable sources in energy production [1]. The Sustainable Development Goals (SDGs) set out by the United Nations highlight renewable energy as key to the success of Agenda 2030 [1]. Possible sources of bioenergy include energy crops, biomass residues and organic wastes. However, direct use of biomass as a heat source may be inefficient and difficult. Complications may arise even at the storage stage, when high humidity is associated with microbiological biomass degradation. The co-combustion of biomass and coal can raise technical and economic issues. Wet biomass may cause instability in the combustion process itself. Incomplete combustion reduces the efficiency of the whole process and leads to energy losses. Incomplete combustion may also make it impossible to maintain the required emission parameters. Given the limited possibilities for using unprocessed biomass, pre-treatment is necessary to improve its energy properties. Various methods of initial biomass preparation are described in the literature, which enable co-combustion with coal in power boilers [2,3]. The process of thermal conversion of biomass to biofuel may include combustion, gasification, biocarbonization, torrefaction, dry distillation and pyrolysis [4,5,6,7,8].

Pyrolysis is a thermochemical treatment, involving the extensive thermal decomposition of organic material under oxygen-limited conditions or in an atmosphere of inert gases. The gas phase contains two kinds of compounds. The first are volatiles that condense and form a dark brown, viscous liquid phase. The second are volatile compounds with low molecular weight (e.g., CO, CO_2_, H_2_, CH_4_ and light hydrocarbons). These non-condensable gases remain in the gas phase. The physical process and chemical reactions that occur in pyrolysis are highly complex, and both the conditions of pyrolysis and the organic matrix (which may originate from different sources) affect the quality of the biochar and its eventual properties. These parameters may be helpful when ranking waste materials as potential sources of biocarbon, and for assessing their suitability for co-firing. These parameters may also be used to evaluate the possibility of using condensing and non-condensing gas products for energy generation.

The aim of this research was to convert biomass into a biofuel with properties similar to those of coal. We used waste from the agricultural industry and municipal management as feedstock. The properties of biochars obtained by biomass carbonization were determined in single-factor experiments. We also characterized the main products of the gas products and condensates. The results could contribute to the development of strategies for biomass treatment and the reduction of emissions, improving the sustainability of biomass conversion processes at an industrial scale.

## 2. Materials and Methods

### 2.1. Materials

Six agricultural waste biomass materials were used in the study: flavored spirits production waste (FSW) (lime, grapefruit and lemon), apple pomace (A.pomace), beetroot pulp (B.pulp), brewer’s spent grain (BSG), bark (pine bark) and municipal solid waste (bark, off-cuts, wood chips, sawdust (MSW)). The analyzed biomasses were pre-prepared by drying and grinding.

### 2.2. Volatile Component Analysis

Volatile component analysis was carried out with use of a GCMS (Gas Chromatography–Mass Spectrometry) (Termo Science Trace GC Ultra) and an RTX—1.60 m × 0.25 mm × 0.25 µm capillary column (Restek, Saunderton, UK), combined with a DSQ-II (Dual-Stage Quadrupole) detector (Thermo Scientific, Austin, TX, USA). All the samples were analyzed in duplicate at a pyrolysis temperature of 550 °C with a heating rate of 20 °C ms^−1^. The samples were collected in Tedlar bags (Merck, Darmstadt, Germany Tedlar^®^ PLV- Push Lock Valve Gas Sampling Bag).

### 2.3. Moisture and Ash Content, Chemical Composition

The total moisture content in the tested biomass was determined using the thermogravimetric approach. The materials were dried at 110 °C until the complete removal of moisture. The ash content was determined using the slow ashing method, in which combustion and annealing of the analytical sample occurs in two stages, differing in temperature and duration. Dry ashing was performed in open inert crucibles in a muffle furnace. The samples (1 ± 0.1 g of biomass powdered by a broom mill) were placed in the cold furnace and combustion was performed with a constant increase in temperature up to 500 °C for 30 min. The temperature was gradually increased to 815 °C over 30 min. Complete ashing was achieved after thermal decomposition for 90 min.

The chemical composition (content of carbon, sulfur, nitrogen) was determined using an elementary analyzer (CE Instruments, Milan, Italy.) and Eager 200 software (amlyzer type 2500), using methionine or BBOT (2,5-(bis(5-tert-butyl-2-benzo-oxazol-2-yl) thiophene) as the reference material.

### 2.4. Combustion Heat and Net Calorific Values, Energy Yield

The calorific value was determined using a 6100 compensated jacket calorimeter (Parr Instruments GmbH, Moline, IL, USA). Net calorific values were calculated on the basis of the amount of heat generated during the complete combustion of a mass unit (1 g) of biomass in an oxygen atmosphere using the formula

LHV = 2.326 × (HHV − 91.23 × C_H_) (1)

where LHV is the net calorific value (Lower Heating Value) (J/g), HHV is the combustion heat and C_H_ is the hydrogen content of the sample (%) [9]. The net calorific value (LHV) of biomass is calculated as the heat of combustion reduced by the heat of water evaporation, obtained from the fuel in the process of combustion and from hygroscopic moisture. The energy densification ratio describes the ratio of the HHV of the dry product to the dry raw material.

Energy densification = HHV_biochar_/HHV_biomass_ (2)

The energy yields were calculated using the equation

Energy yield = (mass_biochar_/mass_biomass_) × (HHV_biochar_/HHV_biomass_) × 100% (3)

Mass yields were calculated using the equation

Mass Yield = mass_biochar_/mass_biomass_ (4)

### 2.5. Carbonization Process

Thermal carbonization (pyrolysis) was performed in a CZYLOK reactor (Jastrzebie-Zdroj, Poland; model FCF 2R) modified in the laboratory to enable the collection of pyrolytic gases. An accurately weighed sample was placed in the oven at room temperature. Thermal decomposition was then performed under conditions of limited oxygen access. The temperature in the reactor was gradually increased to 850 °C over 30 min. Pyrolysis was continued for 60 min at a constant temperature. Subsequently, the sample was left in the oven until the temperature fell. The solid residue after the process was weighed and stored for further analysis. The experiments were carried out in triplicate with seven gas collection points (450, 515, 585, 650, 715, 785 and 850 °C).

## 3. Results

### 3.1. Physico-Chemical Properties and Chemical Composition of the Biomass

The results from proximate and elemental analysis of all the waste samples are given in Table 1. The moisture contents of the tested biomasses in the working state were from 9.65% to 16.54%. The lowest water content in the biomass was observed in waste from vodka production, which can be explained by the fact that these wastes had been dried in the factory before being delivered for analysis. The difference in the moisture content (6.02%) between the bark samples and municipal waste was due to the weather conditions under which the biomasses had been processed and then stored.

The content of mineral substances was similar in most of the analyzed biomasses, ranging from 2.57% to 4.37%. An exception was apple pomace, which contained only 1.05% of inorganic components. This value was in line with the data presented in the literature [10,11]. The ash content of MSW was in line with the average values described in the literature for wood biomass, in the range of 0.3–7.4% [12,13]. These results also indicate that the samples were not contaminated with soil. Our values are much lower than those presented in the literature for coal, i.e., 5–45% (8.5–10.5% on average) [13]. The chlorine content in the analyzed biomass was in the range of 0.02–0.20% and such values are consistent with the literature data for woody biomass, the chlorine content of which ranges from <0.005% to 0.057%. In cultivated plants, the chlorine content may even be >1.00% [2,14]. High chlorine content in crops is often associated with the use of potassium fertilizer.

The total sulfur content was highest for agricultural crops, B.pulp 0.75%, which probably results from the application of fertilizers for agrotechnical treatments. The nitrogen content was similar in all the analyzed biomasses (1.35% ± 0.26%) and did not exceed 1.50%, with the exception of BSG for which it was almost four-fold higher. However, this result is in accordance with the literature data, according to which the nitrogen content in crops can be up to 6.45%. The high content of nitrogen in crops is correlated to their high content of proteins, which can represent up to 40% of the dry mass [2].

The bulk density of the analyzed biomasses varied widely, from 130 to 307 kg/m^3^. This was lower than that of coal, which is on average in the range of 800–1000 kg/m^3^. Low bulk density is uneconomical from the point of view of storage and transport. Therefore, biomass pre-treatment such as grinding, pressing or palletization should be considered.

### 3.2. Characterization of Biochars

#### 3.2.1. Morphology

**Figure 1:**
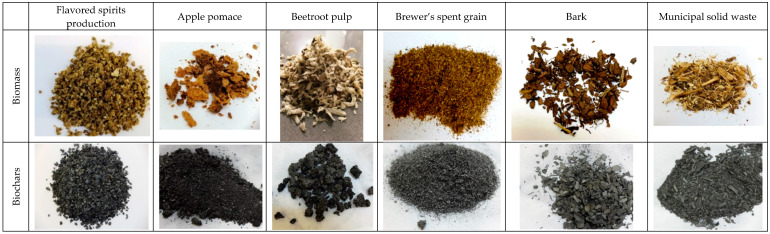
Photographs of the raw and carbonized materials. Thermal decomposition performed under conditions of limited oxygen access with gradually increased temperature up to 850 °C over 30 min and continued for 60 min at a constant temperature.

Applying pyrolysis to the waste biomass resulted in carbonization. Figure 1 presents graphical images of the biomass and the carbonized material. Carbonization occurred in the whole mass, and was as high on the surface as at the core. The transformations throughout the whole volume were probably a consequence of biomass fractionation, generated either by the production process (A.pomace, B.pulp, BSG) or by the grinding applied in this study (MSW, bark, FSW). Carbonization was not followed by major changes in the structure and mass density. The values for most of the carbonizates were similar to those determined for the biomass (Table 1 and Table 2). An increase in mass density occurred only in two samples, apple pomace and MSW. These results are a consequence of pre-treatment, including grinding.

#### 3.2.2. Material and Energy Balance

**Figure 2:**
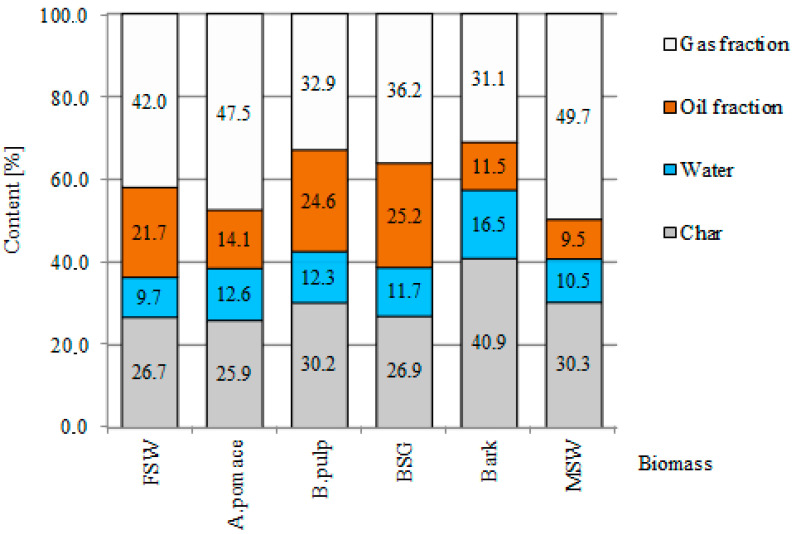
Solid, liquid and gaseous product distribution (wt.%), expressed as average values, obtained with carbonization (850 °C).

The material balances after pyrolysis were investigated using the gravimetric method. The results are presented in Figure 2. The solid residue (i.e., biochars) represented from 26.65% to 40.85% of the initial weight of the organic substance. However, this wide variation in yield was caused mainly by one sample, the bark. When the highest value is excluded, the values were very close, with a mean value of 27.98% ± 2.08%. The decrease in mass can be attributed to two causes. The first is moisture loss and the second is organic matter decomposition, with the formation of volatile products such as CO, CO_2_, CH_4_ and many others. During the process of carbonization, variable amounts of liquid and gas products were formed, in proportions that depended on the biomass. Volatile matter, including water and both gas and oil fractions, composed up to 73% of the initial mass of the samples, with the exception of bark, in which these fractions comprised 59.15%. These differences were probably due to the high content of hemicellulose, cellulose and lignin in bark.

The liquid fraction collected by condensation was composed of oil and water. Based on the mass of the liquid fraction, the oil fraction was calculated by diminishing the weight of the condensate by the moisture content. In general, the content of water was lower in the biomass from fruit waste (9.65% FSW, 12.56% A.pomace). The highest value was obtained for biomass from bark (16.54%). The greatest changes in the amounts of volatile compounds were recorded during heating up to 450 °C.

Table 2 presents the results of proximate and elemental analysis of the biochars obtained from pyrolysis. The carbonization process increased the carbon content on average 1.7-fold, from an average percentage of 46.09% ± 3.65% for biomass to an average percentage of 74.72% ± 5.36% for carbonizates. As a consequence, the C/H ratio also increased, reaching a value seven times higher.

Ash content reached values from 5.38% for A.pomace to 16.95% for B.pulp. Relative to the ash content in the raw materials, these values represent increments of 4.33% and 14.02%, respectively. Similar results from the same thermochemical processes have been described by other authors, using municipal solid waste and biomass [15,16]. High ash biochars are of limited use as fuels in the combustion process, since they can cause excessive ash deposition or slag and contamination phenomena, leading to operational difficulties.

Differences in the elemental compositions of the biomasses and biochars resulting from carbonization clearly illustrate the effect of fuel refining, which involves the removal of water and the elimination of oxygen in the form of oxidized volatile compounds through decarboxylation, decarbonization and dehydration reactions.

To determine the energetic yield of the analyzed process, the energy densification ratio and energy yield were calculated (Equations (2) and (3)). The energy densification ratio, indicating the elevation in HHV during carbonization, differed in a narrow range from 1.372 to 1.724, with a mean value of 1.5615 ± 0.124. However, energy densification does not indicate true changes in the energy value of the product, because it does not take into account the reduction in mass that occurs as a result of the process. When mass loss is considered, greater differences between the biomasses are revealed. The energy yield varied from 36.9% to 61.1% for BSG and bark, respectively. The mean value of the energy yield was 46.97% ± 8.572%. The biochars resulting from carbonization had a net calorific value of between 27 and 32 MJ/kg. These values are comparable or even higher than good-quality milled coal (eco pea coal 24–26 MJ/kg, grain diameter 5–25 mm, produced from specially selected hard coal species to obtain fuel with low contents of sulfur and ash).

#### 3.2.3. Composition Analysis of Non-Condensable Gases

**Figure 3:**
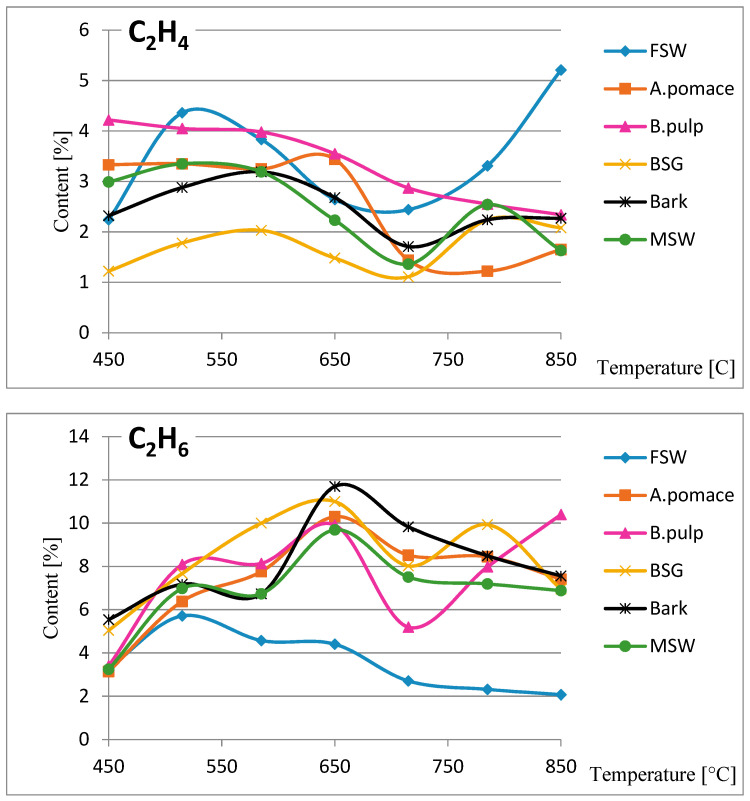

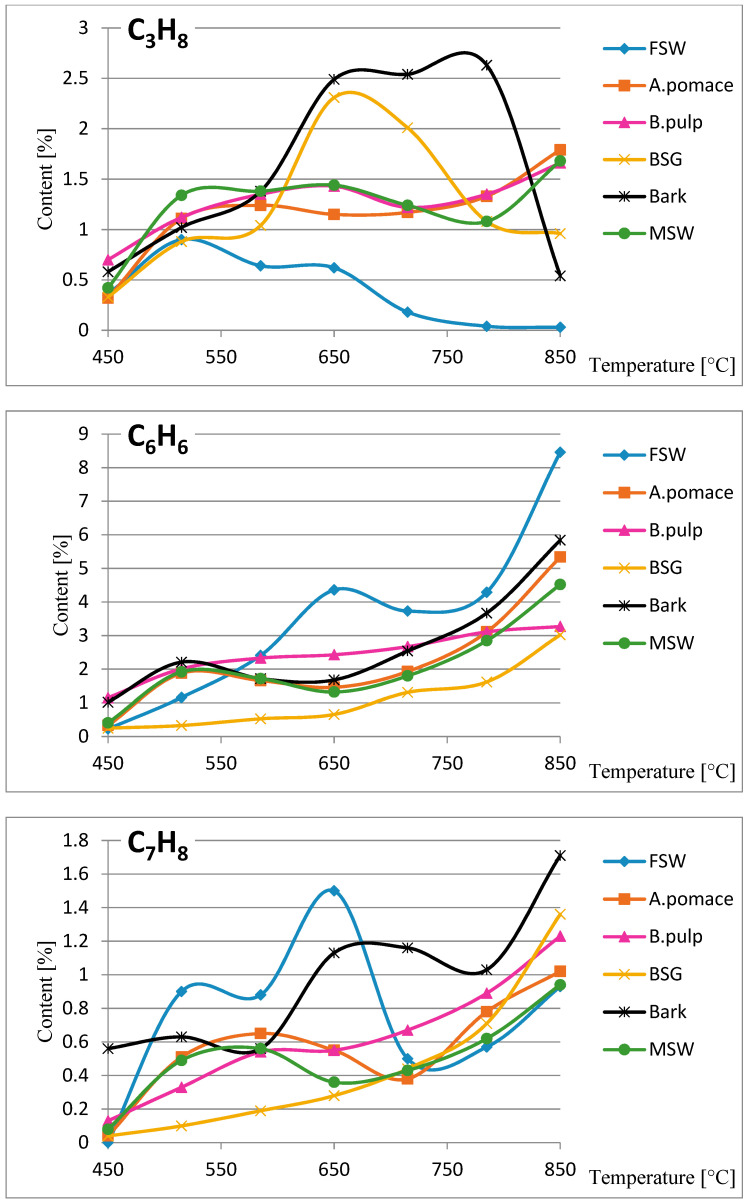
Volatile fractions produced during biomass pyrolysis as a function of temperature. FSW—flavored spirits production waste (lime, grapefruit and lemon), B.pulp—beetroot pulp, A.pomace—apple pomace, BSG—brewer’s spent grain, bark, MSW—municipal solid waste.

Gaseous products are released during pyrolysis by the evaporation or thermal decomposition of the raw material. The amounts of emissions produced during thermal decomposition depend on the composition of the raw material, the heating rate, the temperature and the residence time. Table 3 shows the variations in the content and composition of volatile fractions. Figure 3 shows the variations in the content of hydrocarbons. The major gases produced from biomass carbonization were carbon dioxide and carbon monoxide. The content of CO_2_ decreased gradually, whereas the content of carbon oxide increased with the pyrolysis temperature (i.e., the time of the process), which is assumed to be the result of thermal decomposition in an oxygen-poor atmosphere. The high content of carbohydrates, cellulose, hemicellulose and lignin in the tested materials implies the formation of carbon dioxide, carbon monoxide and water, as a result of decarboxylation, decarbonization and dehydration reactions during thermal decomposition. More CO is produced at elevated temperatures and with longer residence time. The formation of CO is strongly affected by secondary reactions of low molecular weight products (especially aldehyde-type compounds) and CO_2_ is presumably produced in the early stage of cellulose pyrolysis, primarily in decarboxylation reactions [17,18,19].

The yields of N_2_ and CH_4_ were much lower than those for CO and CO_2_. Production of N_2_ was the highest in the initial part of the process of carbonization. At temperatures from 515 °C, only slight fluctuations were observed. There was also a small amount of methane, the concentration of which practically did not change at temperatures above 515 °C. Methane may be formed by methanation (the reaction of carbon with hydrogen oxide to obtain methane and water) at higher temperatures [20].

Based on the chemical compositions of the non-condensing gases, the values for combustion heat and net calorific value were calculated (Table 4). The LHV of the biomass increased with rising pyrolysis temperature. The most intense increase of LHV was observed in the first phase of the process, when the temperature reached 515 °C. Bark and BSG were exceptions, in that a steady increase in LHV was observed up to 650 °C. Further temperature changes up to 715 °C did not have a major effect on LHV, which remained stable with some fluctuations. This was due to the pyrolysis reaction that occurred at higher temperatures. Following this stage of relative stabilization, LHV increased slightly again. These changes were the least pronounced in the case of bark and BGS. In the process of biomass thermal treatment, the major energy loss due to the release of volatile products took place in the torrefaction phase (up to 450 °C). The results for LHV show that non-condensing volatile products can be a valuable source of energy.

#### 3.2.4. Composition Analysis of Condensable Gases—Liquid Products

The color and consistency of the condensates varied depending on the biomass. Images of the fractions are shown in the Supplementary Data (Figure S1). The condensates collected after A.pomace carbonization were distinctive. Their color was lighter and they did not look oily. Liquid phases collected from FSW and MSW were clearly darker than the others. They were oily and with a thicker consistency. As shown in Figure 2, the yield of condensates varied greatly, from 9.7% to 25.2% for MSW and BSG, respectively. The composition of the condensate fractions from the thermal decomposition of biomass appeared to be very complex. Gas chromatography (GC) analysis revealed the presence of over 250 organic compounds (Figure S2, Supplementary Data). The main compounds were cresols, phenols, aromatic hydrocarbons (toluene, benzene, xylene), nitrous aromatic hydrocarbons, aliphatic ketones, furan derivatives and aromatic polycyclic hydrocarbons. The cresol and phenol contents were similar, ranging from 5.2% to 9.2% and from 5.3% to 8.7%, respectively. The total content of benzene, toluene and xylenes ranged from 2.4% to 7.1%. None of the organic compounds from the remaining groups occurred in quantities greater than 1%. Polycyclic aromatic hydrocarbons were probably the product of the condensation of aromatic compounds, forming polycyclic structures [21]. The chemical pathways for cellulose pyrolysis and decomposition have been studied extensively [19,22,23]. According to the systematic review presented by Shen and Gu [19], furan and its furan derivatives are formed as a result of direct ring-opening and rearrangement reactions in cellulose molecules. As our results show, the condensates were composed of different organic compounds that can be recirculated and used as additives for other fuels, producing a positive impact on the environment. The elemental chemical composition values for bio-oils have been extensively reported in the literature [24]. The typical ranges for the products of fast pyrolysis are 50–60% for carbon, 6–9% for hydrogen, 30–40% for oxygen, <0.5% for nitrogen and <0.05% for sulfur [25].

## 4. Conclusions

Carbonization of agricultural waste biomass can be used to generate products with the composition and properties of alternative fuels. In this study, the thermochemical decomposition of waste biomaterials during rapid pyrolysis resulted in homogeneous biochar with yields of up to nearly 41% of the original mass. The biochars had much higher combustion heat and calorific values compared to the biomasses from which they were made. This was due to the fact they no longer contained water, which is physically and chemically bound to these biomasses and alternates in its chemical composition into products with higher carbon content. The biomass transformation process applied in this study enabled the moisture content to be lowered to such an extent that the addition of products no longer had a negative impact on the stability of the combustion process or the total combustion. As a consequence, the efficiency of the entire process was not affected. Both the non-condensable and condensate gaseous products were composed of various organic compounds with sufficiently high combustion heat and net calorific values that they could be recycled and used as additives in other fuels.

## Figures and Tables

**Table 1:** Compositional and elemental analysis of biomass, expressed as average values (±standard deviation). FSW—flavored spirits production waste (lime, grapefruit and lemon), B.pulp—beetroot pulp, A.pomace—apple pomace, BSG—brewer’s spent grain, bark, MSW—municipal solid waste.

Biomass	Moisture Content	Ash	Values of Combustion Heat	Net Calorific Values	Mass Density	Elemental Analysis					
						C	H	N	S	O	Cl
	(%)	(%)	(kJ/kg)	(kJ/kg)	(kg /m^3^)	(%)					
FSW	9.65	3.85 ± 0.34	16.142 ± 600	14,904.3 ± 553.8	243.7 ± 10.0	41.94	5.83	1.32	0.42	37.24	0.09
A.pomace	12.56	1.05 ± 0.01	21.229 ± 135	19,775.2 ± 125.8	155.2 ± 9.3	51.39	6.85	1.44	0.30	36.64	0.07
B.pulp	12.33	2.93 ± 0.02	16.372 ± 28	15,169.2 ± 25.8	155.3 ± 3.0	41.83	5.67	1.32	0.75	35.50	0.20
BSG	11.74	3.59 ± 0.06	20.288 ± 59	18,912.8 ± 55.3	290.1 ± 11.6	46.92	7.12	5.45	0.10	25.83	0.08
Bark	16.54	4.37 ± 0.10	19.523 ± 551	18,477.1 ± 521.5	126.4 ± 7.2	47.23	6.30	1.18	0.18	39.94	0.03
MSW	10.52	2.57 ± 0.19	18.744 ± 104	17,407.3 ± 96.8	130.2 ± 10.1	47.23	6.30	1.18	0.15	40.49	0.02

**Table 2:** Compositional and elemental analysis of biochar, expressed as average values (±standard deviation). FSW—flavored spirits production waste (lime, grapefruit and lemon), B.pulp—beetroot pulp, A.pomace—apple pomace, BSG—brewer’s spent grain, bark, MSW—municipal solid waste, d.m. —dry mass.

Biochars	Mass Yield	Ash	Values of Combustion Heat	Net Calorific Values	Mass Density	Energy Densification Ratio	Energy Yield	Elemental Analysis		
								C	H	N
	(%)	(%)	(kJ/kg)	(kJ/kg)	(kg d.m./m^3^)		(%)	(%)		
FSW	26.65	13.44	27.836 ± 78	26.598 ± 75	238.9 ± 4.9	1.724	46.0	71.47	1.50	1.61
A.pomace	25.89	5.38	32.402 ± 176	30.948 ± 168	218.0 ± 6.9	1.526	39.5	80.18	1.82	2.08
B.pulp	30.18	16.95	26.775 ± 146	25.572 ± 139	120.9 ± 10.9	1.635	49.4	68.54	1.19	1.87
BSG	26.92	14.43	27.840 ± 26	26.465 ± 25	240.3 ± 4.8	1.372	36.9	69.75	1.63	6.42
Bark	40.85	9.35	29.188 ± 26	28.142 ± 26	138.2 ± 3.4	1.495	61.1	79.34	1.58	0.52
MSW	30.26	8.51	30.311 ± 215	28.974 ± 206	236.0 ± 3.4	1.617	48.9	79.06	1.62	1.55

**Table 3:** Chemical composition of non-condensable gases produced during the pyrolysis of biomass, expressed as average values. Content of compounds expressed as relative peak area (%) of gases found in the fraction. C_x_H_y_—hydrocarbons, FSW—flavored spirits production waste (lime, grapefruit and lemon), B.pulp—beetroot pulp, A.pomace—apple pomace, BSG—brewer’s spent grain, bark, MSW—municipal solid waste.

Biomass	Product	450	515	585	650	715	785	850
		(°C)						
FSW	H_2_	0.37	0.81	0.89	1.11	0.42	0.23	0.10
	N_2_	7.34	2.60	2.83	4.14	4.46	3.54	5.51
	CO	35.82	39.9	39.46	35.29	38.34	43.49	42.95
	CO_2_	43.08	27.91	27.54	25.51	31.95	26.84	17.42
	CH_4_	6.33	14.69	16.16	19.06	14.62	15.06	16.52
	CxHy	6.12	13.03	12.33	14.29	9.55	10.52	16.70
A.pomace	H_2_	0.52	1.03	1.77	3.66	2.87	1.11	0.87
	N_2_	9.34	5.44	4.11	4.34	3.53	4.62	4.51
	CO	28.22	22.49	23.20	23.88	32.27	35.63	39.85
	CO_2_	44.78	42.23	40.44	35.37	32.00	26.66	20.34
	CH_4_	8.87	14.66	15.11	15.22	15.33	16.66	17.01
	CxHy	7.16	13.23	14.56	16.98	13.44	14.89	17.21
B.pulp	H_2_	1.01	1.46	2.45	4.66	2.11	2.01	1.13
	N_2_	7.44	4.23	4.32	4.34	4.67	4.87	4.55
	CO	19.59	13.44	12.57	13.95	24.36	24.86	22.39
	CO_2_	50.66	48.78	45.67	39.76	35.44	30.35	29.88
	CH_4_	10.6	15.38	17.66	18.67	20.11	21.46	22.65
	CxHy	9.47	15.60	16.34	17.87	12.62	15.88	18.96
BSG	H_2_	0.66	1.89	2.22	3.13	3.45	2.04	0.84
	N_2_	7.74	4.21	4.41	4.35	4.76	3.45	3.02
	CO	28.09	29.33	30.55	31.95	34.57	37.59	43.33
	CO_2_	49.78	42.05	36.76	30.64	29.94	25.98	23.55
	CH_4_	6.25	10.07	11.01	12.15	12.77	13.53	13.88
	CxHy	6.88	10.76	13.84	15.77	12.89	15.58	14.37
Bark	H_2_	0.56	0.72	1.75	2.14	2.35	1.82	0.84
	N_2_	7.32	3.71	4.05	3.15	3.40	3.86	3.23
	CO	26.21	35.11	29.54	27.85	28.72	32.75	38.37
	CO_2_	43.21	31.65	28.77	25.00	25.94	23.06	20.29
	CH_4_	11.08	14.28	17.84	19.8	20.99	19.58	18.26
	CxHy	10.02	13.92	16.59	19.66	17.78	18.06	17.92
MSW	H_2_	0.64	1.27	1.89	2.53	1.06	0.62	0.21
	N_2_	8.97	3.51	3.41	3.27	3.76	4.02	4.5
	CO	29.25	33.34	31.75	30.89	33.84	37.59	40.59
	CO_2_	46.32	34.23	34.12	31.26	31.95	25.98	21.30
	CH_4_	7.25	13.25	14.78	15.85	15.23	16.29	16.87
	CxHy	7.14	14.08	13.58	15.04	12.34	14.28	15.65

**Table 4:** Net calorific value (Lower Heating Value) (LHV) of non-condensable gases from pyrolysis of the analyzed biomasses, expressed as average values. C_x_H_y_—hydrocarbons, FSW—flavored spirits production waste (lime, grapefruit and lemon), B.pulp—beetroot pulp, A.pomace—apple pomace, BSG—brewer’s spent grain, bark, MSW—municipal solid waste.

	Biomass					
Temperature	FSW	A.pomace	B.pulp	BSG	Bark	MSW
°C	MJ/Nm^3^	MJ/Nm^3^	MJ/Nm^3^	MJ/Nm^3^	MJ/Nm^3^	MJ/Nm^3^
450	10.905	11.589	13.388	10.512	15.135	11.284
515	20.552	18.746	19.302	14.892	20.983	20.253
585	21.471	19.942	21.114	17.628	21.088	20.286
650	24.939	21.475	22.993	20.115	26.394	21.112
715	19.507	20.565	21.481	19.486	26.486	19.806
785	21.333	23.565	24.708	21.909	27.338	22.745
850	29.170	27.729	27.259	23.568	29.158	25.970

## Supplementary Materials

The following are available online at https://www.mdpi.com/1996-1944/13/21/4971/s1, Figure S1: Photograph of the condensable gases—liquid products collected after pyrolysis of agricultural waste biomass. FSW—flavored spirits production waste (lime, grapefruit and lemon); B.pulp—beetroot pulp; A.pomace—Apple pomace; BSG—brewer’s spent grain; bark; MSW—municipal solid waste., Figure S2: GC–MS separation of organic compounds extracted to chloroform from the condensates obtained by carbonization of biomass. FSW—flavored spirits production waste (lime, grapefruit and lemon); B.pulp—beetroot pulp; A.pomace—Apple pomace; BSG—brewer’s spent grain; bark; MSW—municipal solid waste.
