# Supplementary material for: Biochars from Post-Production Biomass and Waste from Wood Management: Analysis of Carbonization Products
Source: Materials (Basel). 2020 Nov 4;13(21):4971. doi: 10.3390/ma13214971 (PMC7663828; doi:10.3390/ma13214971)
Supplement: Supplementary file 1 [file materials-13-04971-s001.pdf]

Supplementary Materials:

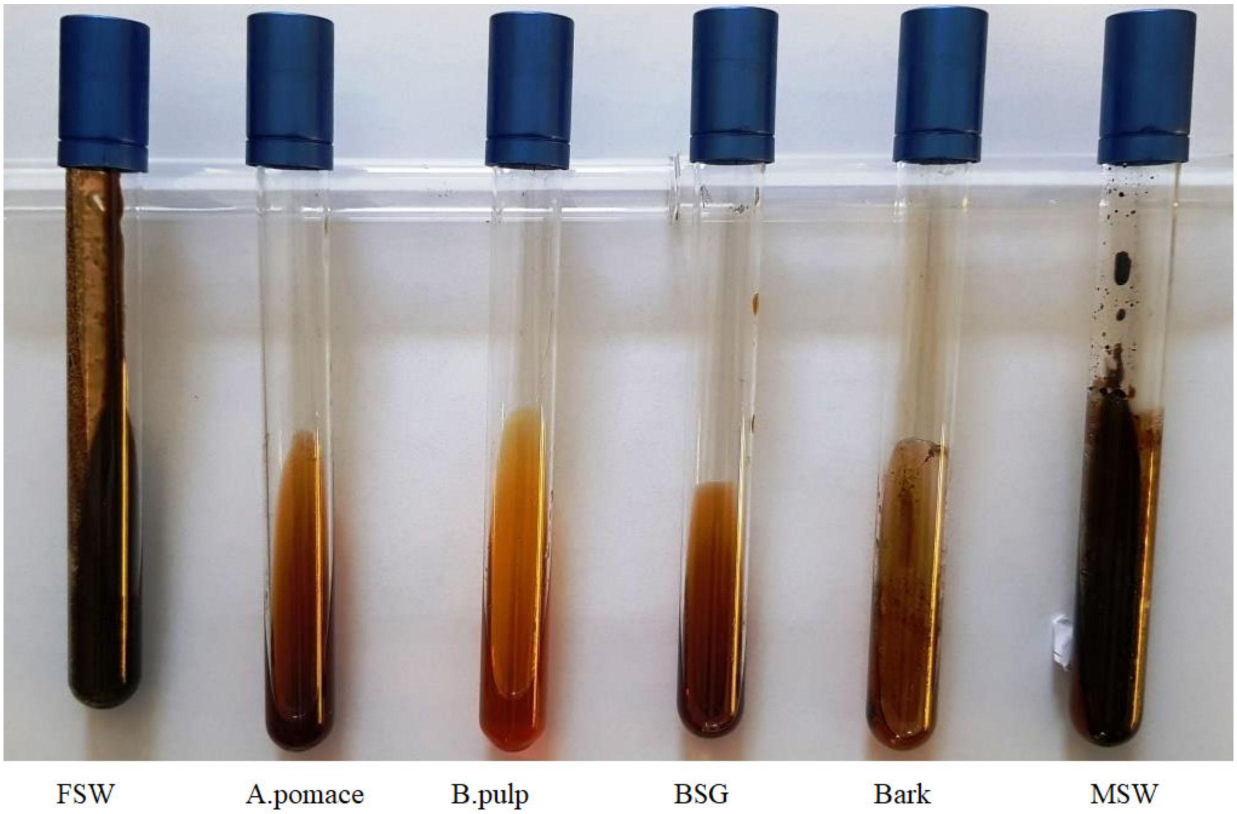

Figure S1. Photograph of the condensable gases—liquid products collected after pyrolysis of agricultural waste biomass. FSW—flavored spirits production waste (lime, grapefruit and lemon); B.pulp—beetroot pulp; A.pomace—Apple pomace; BSG—brewer's spent grain; bark; MSW—municipal solid waste.

FSW

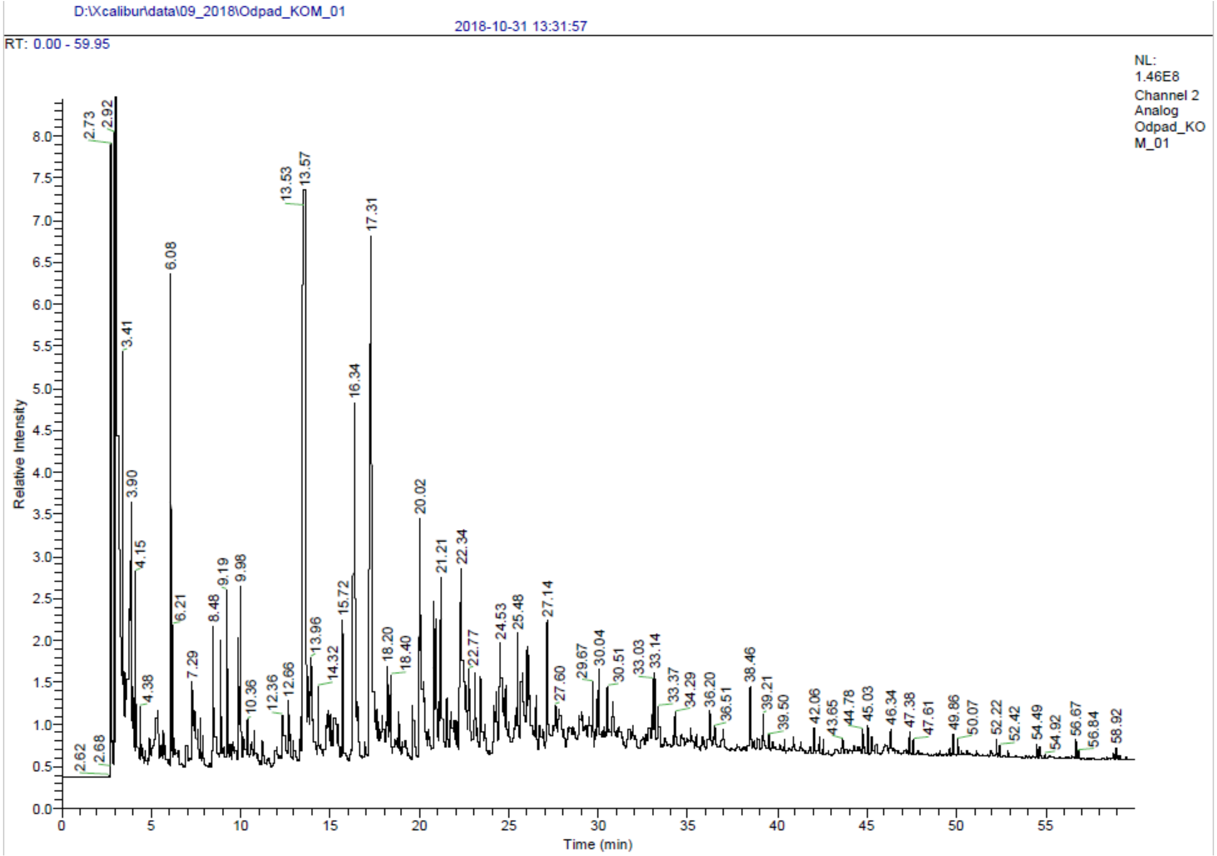

D:\Xcalibur\data\09_2018\Odpad_KOM_01
2018-10-31 13:31:57
RT: 0.00 - 59.95
NL:
1.46E8
Channel 2
Analog
Odpad_KO
M_01
Relative Intensity
Time (min)

A.pomace.

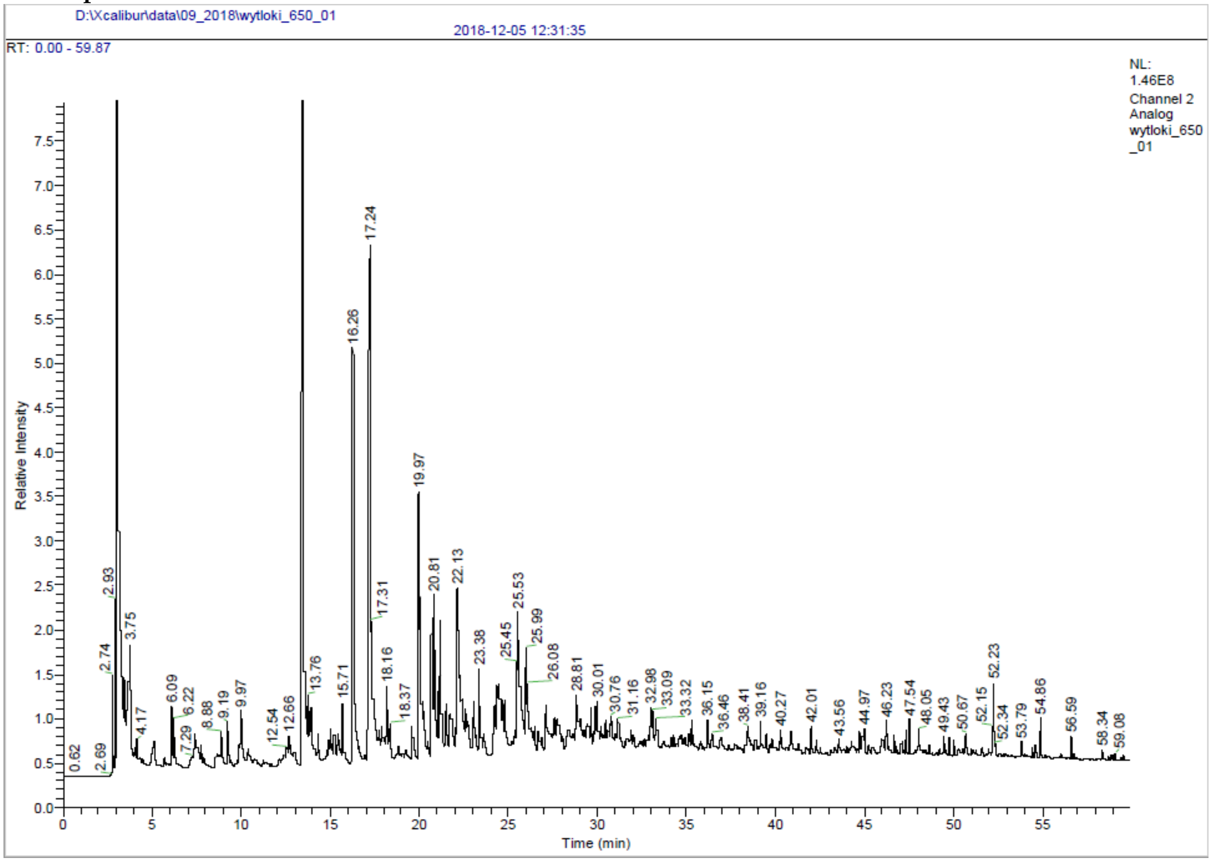

D:\Xcalibur\data\09_2018\wytloki_650_01
2018-12-05 12:31:35
RT: 0.00 - 59.87
NL:
1.46E8
Channel 2
Analog
wytloki_650
_01
Relative Intensity
Time (min)

B.pulp

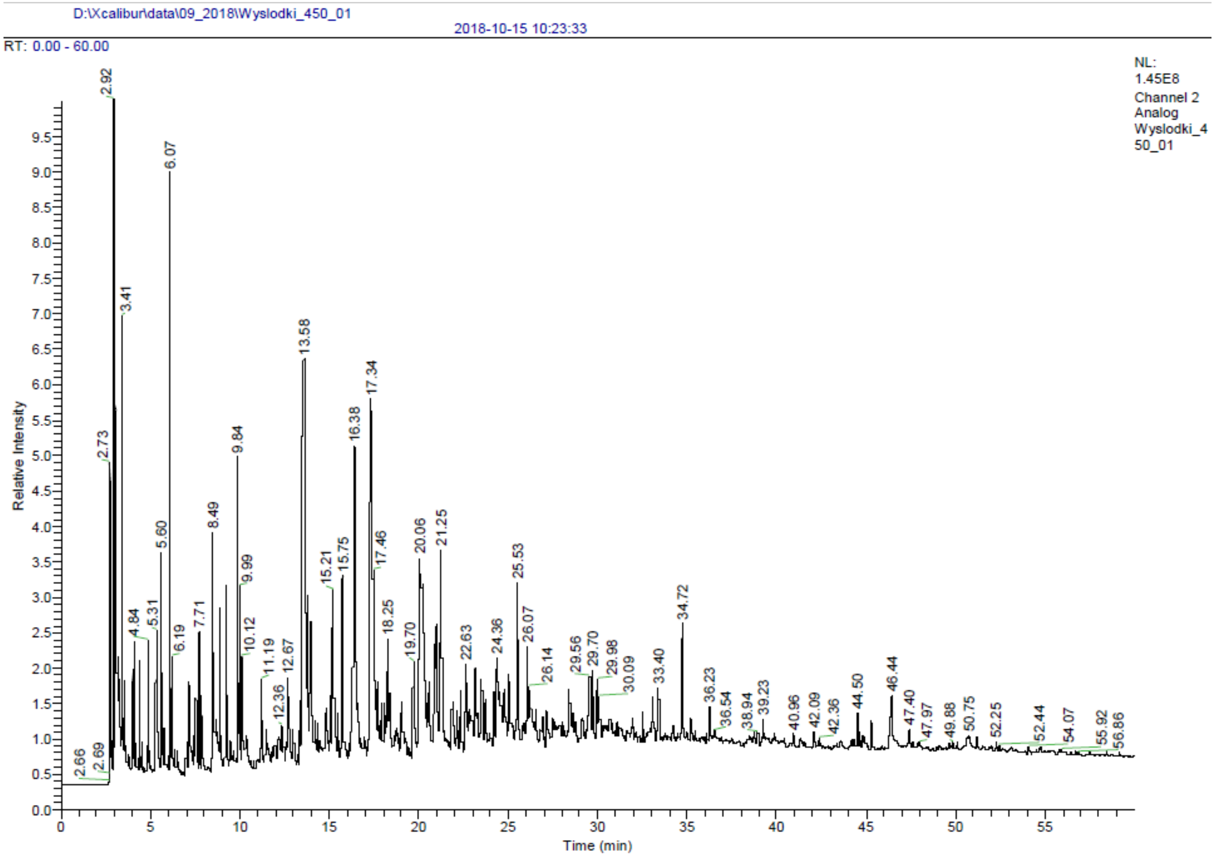
D:\Xcalibur\data\09_2018\Wyslodki_450_01
2018-10-15 10:23:33
RT: 0.00 - 60.00
NL:
1.45E8
Channel 2
Analog
Wyslodki_4
50_01
Relative Intensity
Time (min)
2.92
6.07
3.41
13.58
17.34
16.38
2.73
9.84
8.49
20.06
21.25
5.60
17.46
15.21
15.75
25.53
9.99
34.72
5.31
4.84
10.12
7.71
6.19
18.25
26.07
11.19
12.67
19.70
22.63
24.36
26.14
29.56
29.70
29.98
30.09
33.40
12.36
36.23
36.54
38.94
39.23
40.96
42.09
42.36
44.50
46.44
47.40
47.97
49.88
50.75
52.25
52.44
54.07
55.92
56.86
2.66
2.69

BSG

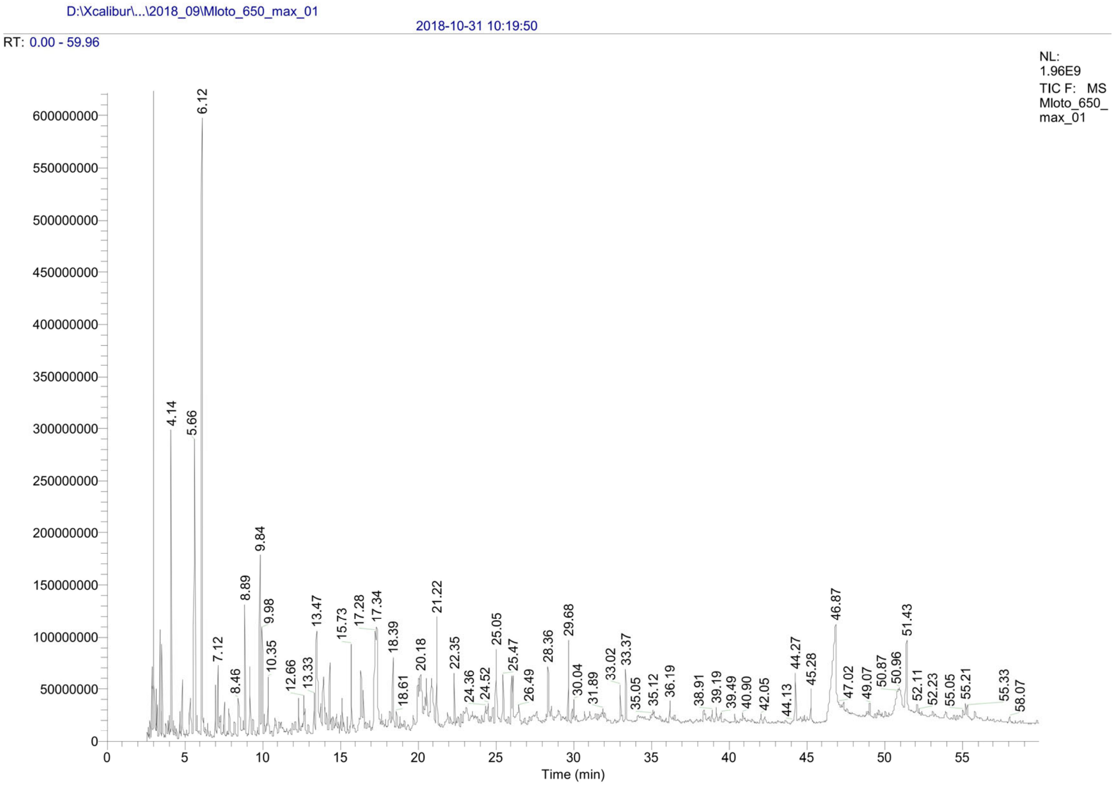
D:\Xcalibur\...\2018_09\Mloto_650_max_01
2018-10-31 10:19:50
RT: 0.00 - 59.96
NL:
1.96E9
TIC F: MS
Mloto_650_
max_01
Time (min)
6.12
4.14
5.66
9.84
8.89
9.98
13.47
15.73
17.28
17.34
18.39
21.22
7.12
8.46
10.35
12.66
13.33
18.61
20.18
22.35
24.36
24.52
25.05
25.47
26.49
28.36
29.68
30.04
31.89
33.02
33.37
35.05
35.12
36.19
38.91
39.19
39.49
40.90
42.05
44.13
44.27
45.28
46.87
47.02
49.07
50.87
50.96
51.43
52.11
52.23
55.05
55.21
55.33
58.07

Bark

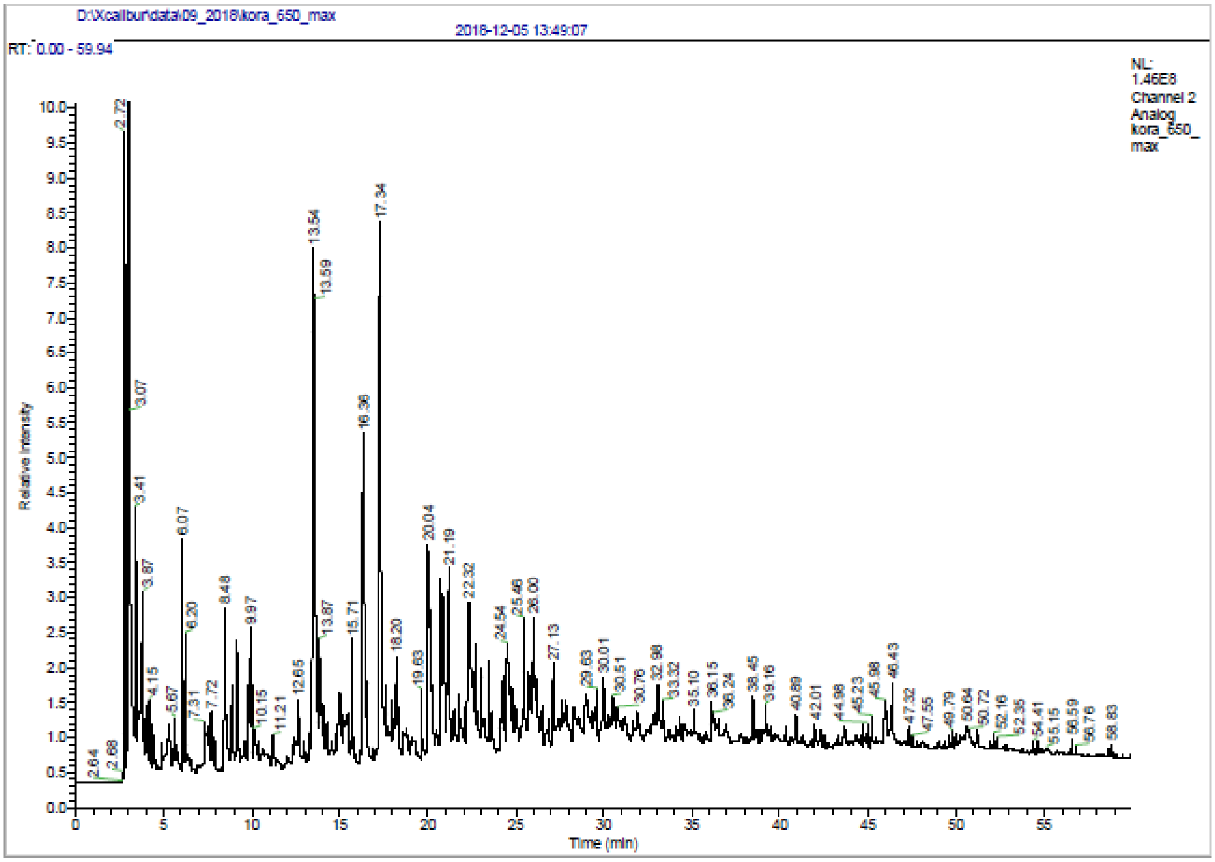
D:\Xcalibur\data\09_2018\kora_650_max
2018-12-05 13:49:07
RT: 0.00 - 59.94
NL:
1.46E8
Channel 2
Analog
kora_650_
max
Relative Intensity
Time (min)
2.72
13.54
17.34
13.59
3.07
16.36
3.41
6.07
20.04
21.19
3.87
8.48
22.32
9.97
25.46
26.00
6.20
13.87
15.71
18.20
24.54
27.13
29.63
30.01
30.51
30.76
32.98
33.32
35.10
36.15
36.24
38.45
39.16
40.89
42.01
44.98
45.23
45.98
46.43
47.32
47.55
49.79
50.64
50.72
52.16
52.35
54.41
55.15
56.59
56.76
58.83
4.15
5.67
7.31
7.72
10.15
11.21
12.65
19.63
2.64
2.68

MSW

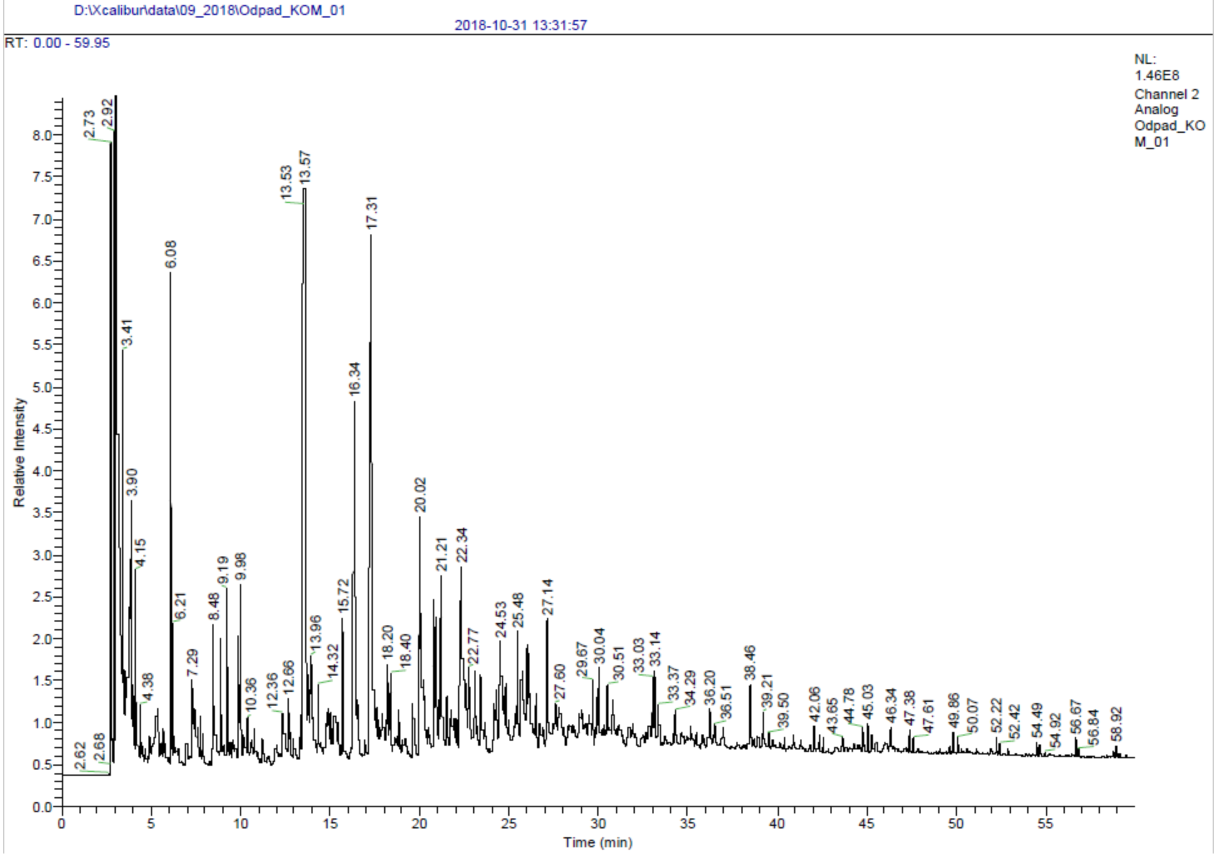
D:\Xcalibur\data\09_2018\Odpad_KOM_01
2018-10-31 13:31:57
RT: 0.00 - 59.95
NL:
1.46E8
Channel 2
Analog
Odpad_KO
M_01
Relative Intensity
Time (min)
2.73
2.92
13.53
13.57
17.31
6.08
3.41
16.34
3.90
20.02
4.15
22.34
21.21
9.19
9.98
8.48
6.21
15.72
13.96
14.32
18.20
18.40
22.77
24.53
25.48
27.14
27.60
29.67
30.04
30.51
33.03
33.14
33.37
34.29
36.20
36.51
38.46
39.21
39.50
42.06
43.65
44.78
45.03
46.34
47.38
47.61
49.86
50.07
52.22
52.42
54.49
54.92
56.67
56.84
58.92
7.29
4.38
10.36
12.36
12.66
2.62
2.68

Figure S2. GC–MS separation of organic compounds extracted to chloroform from the condensates obtained by carbonization of biomass. FSW—flavored spirits production waste (lime, grapefruit and lemon); B.pulp—beetroot pulp; A.pomace—Apple pomace; BSG—brewer's spent grain; bark; MSW—municipal solid waste.
